# Supplementary material for: Risk of obstetric anal sphincter tear among primiparous women with a history of female genital mutilation, giving birth in Sweden
Source: PLoS One. 2022 Dec 30;17(12):e0279295. doi: 10.1371/journal.pone.0279295 (PMC9803268; doi:10.1371/journal.pone.0279295)
Supplement: Attachment — Submitted filename: Response to reviewers.docx [file pone.0279295.s001.docx]

Response to the reviewer (PLOS ONE)

**Risk of obstetric anal sphincter tear among primiparous women with a history of female genital mutilation, giving birth in Sweden**

Dear Editor

We appreciate you and the reviewer for your time in reviewing our paper and providing valuable comments. The authors have carefully considered the comments and tried our best to address every one of them. We hope that the manuscript after careful revisions meets your high standards. The authors welcome further constructive comments if any. Below we provide the point-by-point responses. All modifications in the manuscript have been highlighted in yellow.

Sincerely

Bita Eshraghi, MD

**1.Since the study includes a large amount of women in the cohort, there are a broader range of statistical possibilities. First, it is important to evaluate many different possible risk factors of OASI and include them in the multivariable analyses. The authors only include a few, and they do not report why they present different models. I would suggest that more variables are added (for example maternal age, ethnicity, etc) and that the authors evaluate the risk of interaction between the different factors. Especially, the interactions between FGM, OASI and episiotomy is interesting.**

Thank you for your comment on this issue.

The risk factors for OASI have been discussed over time. We chose to use the risk factors with strongest evidence (line 94-98), that is, nulliparous, birthweight, instrumental delivery, ethnicity and in addition episiotomy. We had undermined the major risk factors for OASI and had only extracted those variables from the register.

As suggested, maternal age is included in all models in table 4 and 5. In a sensitivity analysis (table 5), women born in countries with high prevalence of FGM were examined separately to disentangle the potential confounding effect of country of origin on risk of our outcome. We chose to present table 4 and 5 with 4 different models to show the reader how the different variables affect the outcome.

We have added a section regarding interactions in page 8, line 166-167 and page 12, line 195-197

**2. Indication of Episiotomy. When and how is FGM diagnosed and registered, when and how is OASIS diagnosed**

Thank you for pointing out the need for clarification regarding indication for episiotomy, and how and when OASI and FGM are diagnosed. This has given us the opportunity to improve the manuscript through revision. We have now added 3 sections in which we have explained this.

Page 14, line 235-238.
Page 6, lines 125-126.
Page 7, lines 146-149.

**3. Are the any codes in the used dataset, that evaluates prior to the delivery to enable vaginal birth without extensive tearing or cutting**

As described in line 248-250 the code for defibulation (TLF00) was first introduced in 2019 and since our data covers 2014-2018, we could not use this code.

**4. Based on the finding, do the authours suggest a modification of guidling women with a history of FGM regarding mode of delivery?**

Thank you for your comment.

Based on the results from our study we suggest that national guidelines on obstetric management also includes specific awareness regarding the risk for OASI that women with a history of FGM have. In addition, these women should always be given the choice to undergo a defibulation antenatal or intrapartal.

We have added this paragraph to the discussion section on page 17, line number 314-318.

**5. Line 284-285, the authors state that scarring of the labia and the clitoral glans should not increase the risk of OASI. However, they do not come up with an alternative biomechanical explanation for the found association? What about the elasticity in the scarred tissue? What about preventive strategies, like the Finnish grip, warm cloth etc. Please elaborate further on this.**

Thank you for your comment on this issue. We have now a section in which we have explained this. Other preventive strategies such as Finnish grip or warm cloth are considered routine practice in Sweden and were not specifically registered in this study.

Page 16-17, line number 298-309

In addition, we change the word risk to association since this would be the more correct term.
Line number: 234-235
Line number: 265-266

We also added a limitation in the abstract
Line: 38-39

We also did some clarification in data report on line number: 116
