# Supplementary material for: Risk of obstetric anal sphincter tear among primiparous women with a history of female genital mutilation, giving birth in Sweden
Source: PLoS One. 2022 Dec 30;17(12):e0279295. doi: 10.1371/journal.pone.0279295 (PMC9803268; doi:10.1371/journal.pone.0279295)
Supplement: Attachment — Submitted filename: Response to reviewers.docx [file pone.0279295.s002.docx]

Response to the reviewers (PLOS ONE)

**Risk of obstetric anal sphincter tear among primiparous women with a history of female genital mutilation, giving birth in Sweden**

Dear Editor

We appreciate you and the reviewers for your time in reviewing our paper and providing valuable comments. All authors have carefully considered the comments and have tried our best to address every one of them. We hope that the manuscript after our revisions meets your high standards, and we welcome further constructive comments if any. Below we provide the point-by-point responses. All modifications in the manuscript have been highlighted in yellow.

Sincerely

Bita Eshraghi, MD

**1.In my opinion authors have to improve the paper referring in the text if the pregnancy came from IVF or natural conception and stress how is important the impact of assisted reproduction techniques on the neuro-psycho-motor outcome of newborns and about eventually congenital infections.**

Thank you for your comment on this issue. Our main purpose has been to investigate the association between FGM and obstetric anal sphincter tear. New-borns neuro-psycho-motor outcome was not included in our analysis, and we also have no information about the conception mode or potential infections prior to delivery. Further, we do not have ethical permission to extract information regarding infections and mode of conception.

**2. Regarding lines 133-136, I would suggest adding to the limitation that this coding weakness may lead to the inclusion of non-Swedish women who received vulvar surgery for precancerous indications but have them classified as patients with FGM. This could lead to overestimation of the chance of having OASI in patients with FGM.**

The coding could cause an overestimate in the FGM group, however, the mean age of vulva cancer is around 70 years old, so the risk is very low among women in fertile age. Only 22 cases had the Z907 code in the cohort.
There is however, un underestimation of FGM coding due to insufficient reporting of FGM in the medical records that probably leads to an underestimation of the risk of OASIS.

**4. In line 159, please provide a reference that justifies the consideration of a p value equals to 0.05 as statistical significance difference.**

The significance level var chosen because similar studies have used the same level of significance. Unfortunately, there was a mistake in the manuscript referring to P value being equal to 0.05. This in now corrected.

**7.** **Please include a data availability statement to comply with journal’s policy**

All data is now available. We added the p-value to line 196.

All other comments regarding missing words etc have been corrected.
